# Photoluminescence Study of the Photoinduced Phase Separation in Mixed-Halide Hybrid Perovskite CH_3_NH_3_Pb(Br_x_I_1−x_)_3_ Crystals Synthesized via a Solvothermal Method

**DOI:** 10.1038/s41598-017-18110-6

**Published:** 2017-12-18

**Authors:** Baohua Zhang, Fuqiang Guo, Junjun Xue, Lianhong Yang, Yafei Zhao, Mei Ge, Qing Cai, Bin Liu, Zili Xie, Dunjun Chen, Hai Lu, Rong Zhang, Youdou Zheng

**Affiliations:** 1 Key Laboratory of Advanced Photonic and Electronic Materials, School of Electronic Science and Engineering, Nanjing University, Nanjing, 210093 China; 2 Department of Physics, Changji College, Changji, 831100 China; 3 School of Electronic Science and Engineering, Nanjing University of Posts and Telecommunications, Nanjing, 210023 China

## Abstract

We systematically synthesized mixed-halide hybrid perovskite CH_3_NH_3_Pb(Br_x_I_1−x_)_3_ (0 ≤ x ≤ 1) crystals in the full composition range by a solvothermal method. The as-synthesized crystals retained cuboid shapes, and the crystalline structure transitioned from the tetragonal phase to the cubic phase with an increasing Br-ion content. The photoluminescence (PL) of CH_3_NH_3_Pb(Br_x_I_1−x_)_3_ crystals exhibited a continuous variation from red (768 nm) to green (549 nm) with increasing the volume ratio of HBr (V_HBr_%), corresponding to a variation in the bandgap from 1.61 eV to 2.26 eV. Moreover, the bandgap of the crystals changed nonlinearly as a quadratic function of x with a bowing parameter of 0.53 eV. Notably, the CH_3_NH_3_Pb(Br_x_I_1−x_)_3_ (0.4 ≤ x ≤ 0.6) crystals exhibited obvious phase separation by prolonged illumination. The cause for the phase separation was attributed to the formation of small clusters enriched in lower-band-gap, iodide-rich and higher-band-gap, bromide-rich domains, which induced localized strain to promote halide phase separation. We also clarified the relationship between the PL features and the band structures of the crystals.

## Introduction

Organic-inorganic hybrid perovskite MAPbX_3_ (MA: methylammonium; X: halide) materials are potential candidates for use in optoelectronic devices^1^, including lasers^2^, photodetectors^3^, photosensitive transistors^4^, and light-emitting devices (LEDs)^1^. The power conversion efficiencies (PCEs) of organic-inorganic hybrid perovskite solar cells (PSCs) have increased from 3%^5^ to 23.6%^6^ over the past few years due to their unique features, such as broad and strong light absorption^7^, longer carrier lifetime^8^, long charge-carrier diffusion length^9,10^, high carrier mobility^11^ and small exciton binding energy^12^. At the same time, one attractive feature of hybrid perovskites as photovoltaic absorbers is that their bandgap (E_g_) can be tuned continuously in several ways, such as substituting the central organic molecule MA with FA (formamidinium)^13^, replacing Pb with other metals (Sn or Ge)^14^, and alloying different halides into the structure.

In a few short years, the mixed-halide perovskite CH_3_NH_3_Pb(Br_x_I_1−x_)_3_ materials have been successfully produced via substitution of I with Br ions^15–21^, corresponding to a varying bandgap from 1.5 eV to 2.2 eV^14^. These properties make this class of material for use in multi-colour light-emitting^16^ or lasing^22^ applications and for the larger bandgap absorption in tandem solar cells^23^. Tu *et al*.^24^ modulated CH_3_NH_3_Pb(Br_x_I_1−x_)_3_ films, resulting in PCEs exceeding 18% at x = 0.14, which was a significant improvement of the photovoltaic performance. Additionally, the improvement of the open-circuit voltage of CH_3_NH_3_Pb(Br_x_I_1−x_)_3_ solar cells has a strong relationship with the Br-ion content. Although the bandgaps of perovskites can be tuned by introducing bromide ion, there have some unexpected effects also emerged, such as light-induced effects^25–28^. CH_3_NH_3_Pb(I_x_Br_1−x_)_3_ (0.1 < x < 0.8) materials undergo phase separation into iodide-rich and bromide-rich regions under prolonged illumination and revert to their original states after a few minutes in the dark, which leads to the formation of smaller-band-gap “trap states”^25^. Such de-mixing of the halides is detrimental to the photovoltaic performance because it leads to charge-carrier trapping in halide-rich regions, which decreases the open-circuit voltage with an increasing bromide content above 20%^24^. Unfortunately, many mixed-halide perovskite used in solar cells have rarely been tested for their stability regarding phase segregation.

And also to the best of our knowledge, the most common method employed to fabricate mixed-halide CH_3_NH_3_Pb(Br_x_I_1−x_)_3_ materials is to spin-coat solutions of stoichiometric Pb^2+^ and the halides (I^−^ + Br^−^) on a suitable substrate, followed by annealing^4,16,25^. However, this method requires many kinds of organic solvents, long reaction times, or careful adjustment of the reaction conditions, etc. To date, a facile and rapid method is still needed to synthesize mixed-halide perovskite materials. Facile solvothermal methods have been considered as most promising routes ascribed to their advantages of low temperature, a single-step process, and high reproducibility^29^. Zhao *et al*.^30^ also demonstrated a facile synthetic approach for preparing mixed-halide perovskite CH_3_NH_3_Pb(Br_1−x_Cl_x_)_3_ crystals by solvothermal growth. However, CH_3_NH_3_Pb(Br_x_I_1−x_)_3_ crystals synthesized by a solvothermal method have not been investigated to date.

In this study, CH_3_NH_3_Pb(Br_x_I_1−x_)_3_ (0 ≤ x ≤ 1) crystals in the full composition range were synthesized for the first time by a facile solvothermal method. We used X-ray diffraction (XRD) and photoluminescence (PL) measurements to detect the transformation of the phase structure and the variations of the optical properties in the CH_3_NH_3_Pb(Br_x_I_1−x_)_3_ (0 ≤ x ≤ 1) system. At same time, PL was applied to study the photoinduced phase separation in the mixed-halide perovskite CH_3_NH_3_Pb(Br_x_I_1−x_)_3_ crystals. Therefore, more studies are required to understand the fundamental properties in mixed-halide perovskites CH_3_NH_3_Pb(Br_x_I_1−x_)_3_, which will be very important in optimizing related optoelectronic devices.

## Results and Discussion

**Figure 1:**
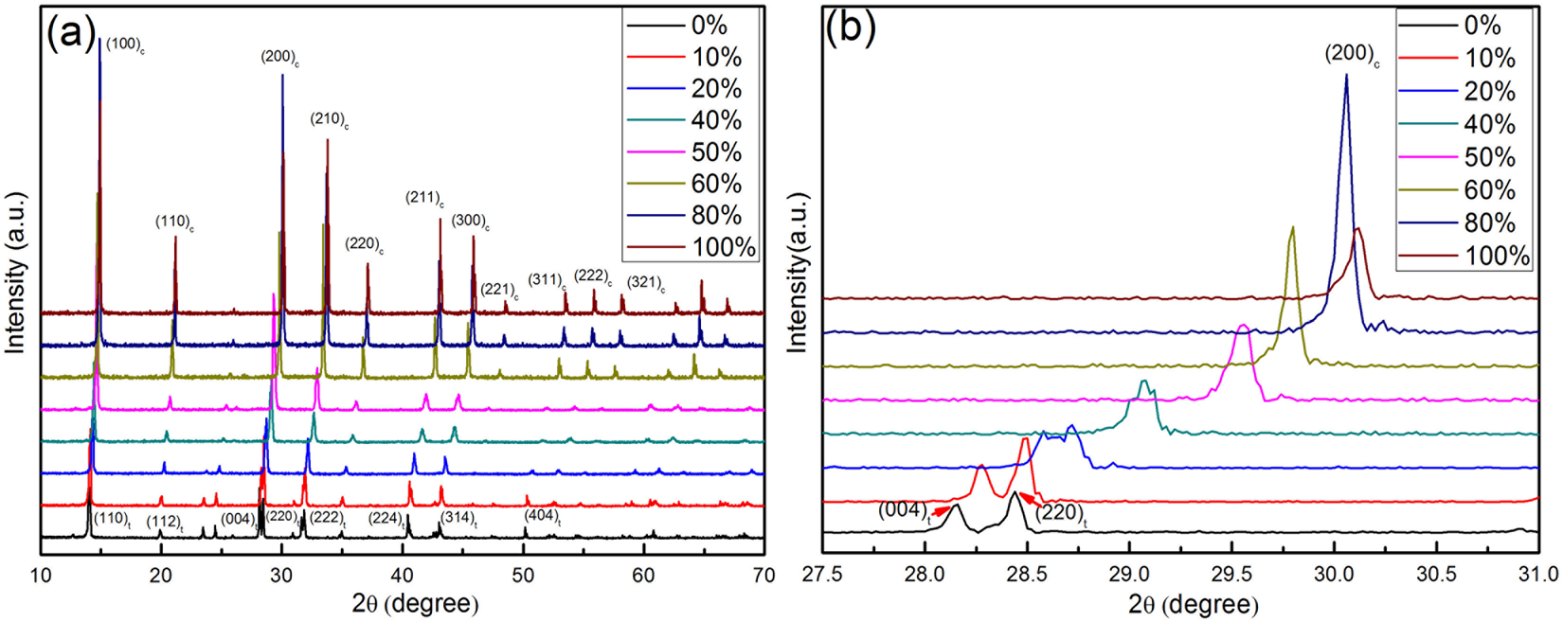
(**a**) The XRD patterns of CH_3_NH_3_Pb(Br_x_I_1−x_)_3_ obtained with V_HBr_% for 0%, 10%, 20%, 40%, 50%, 60%, 80%, and 100%, (**b**) The XRD patterns of CH_3_NH_3_Pb(Br_x_I_1−x_)_3_ magnified in 2θ from 27.5° to 31° (the subscript *c* is defined as cubic phase, and the subscript *t* is defined as tetragonal phase).

To analyse the phase structure transformation of the mixed-halide perovskite crystals with increasing V_HBr_% from 0 to 100%, the XRD patterns of CH_3_NH_3_Pb(Br_x_I_1−x_)_3_ crystals are shown in Fig. 1(a), and the magnified patterns in 2θ = 27.5 −31° are shown in Fig. 1(b). According to the XRD patterns in Fig. 1(a), the CH_3_NH_3_PbI_3_ and CH_3_NH_3_PbBr_3_ crystals with V_HBr_% for 0% and 100% have a tetragonal phase structure with the *I4*/*mcm* space group and a cubic structure phase with the $Pm\bar{3}m$ space group, respectively, which agree with previous reports^20^. The diffraction peaks show a systematic shift to higher scattering angles with increasing V_HBr_%, which indicates a decrease of the unit cell size with increasing bromine content. Because the gradual substitution of the larger I atoms with the smaller Br atoms decreases the lattice spacing. The XRD peaks in Fig. 1(a) are also relatively sharp and no peaks of impurities were detected, thus these materials are good crystals and high purities.

CH_3_NH_3_PbI_3_ (V_HBr_% = 0%) has two peaks located at 28.15° and 28.44°, as shown in Fig. 1(b), which are indexed to (004) and (220) planes of the tetragonal phase. The (004) diffraction peak gradually disappears and finally merges into a single peak upon increasing V_HBr_% above 20%, corresponding to the (200) plane of the cubic phase, which confirms that the symmetry of phase structure improve. Further substitution of I ion with Br ions into the tetragonal phase of CH_3_NH_3_PbI_3_ causes the systematic shift of the (200) peak towards higher scattering angle. In other words, the tetragonal (pseudo-cubic) phase can transform into the cubic phase with increasing V_HBr_%. It ascribe to that the smaller halide ion radius is favourable for the formation of the cubic structure, which is accepted as a criterion for the distortion of the PbX_6_ octahedra^16^.

**Figure 2:**
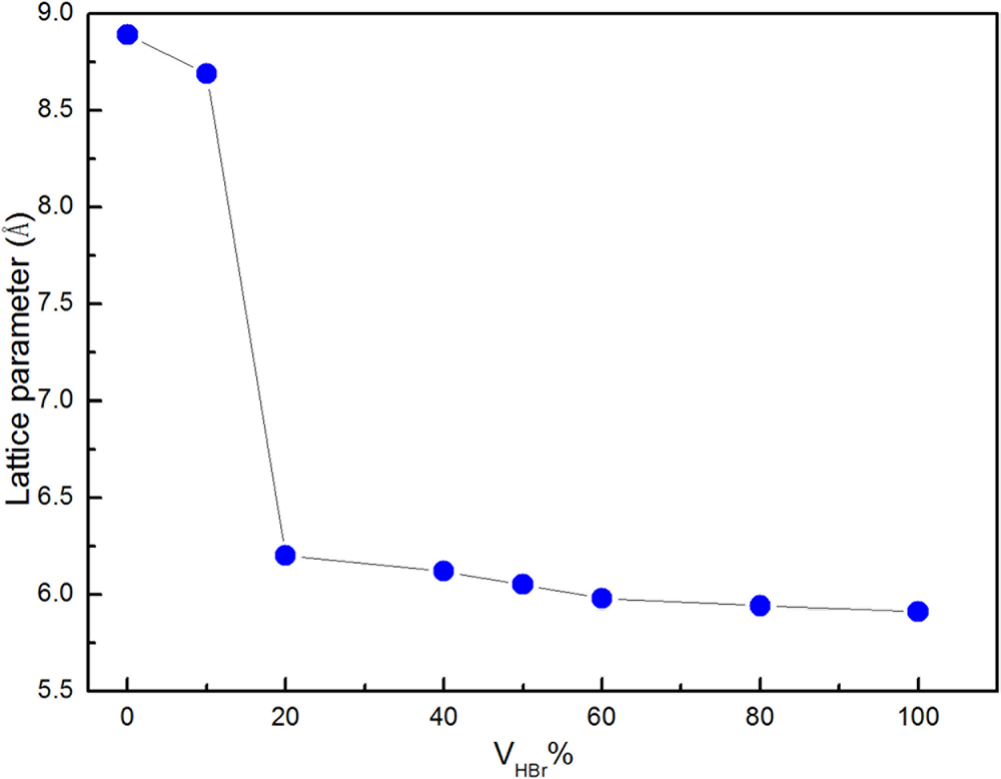
The relationship between the lattice parameter *a* of the CH_3_NH_3_Pb(Br_x_I_1−x_)_3_ crystals and V_HBr_%.

The lattice parameter *a* of the CH_3_NH_3_Pb(Br_x_I_1−x_)_3_ crystals indexed by pseudo-cubic or cubic symmetry as a function of V_HBr_% is shown in Fig. 2. The *a* gradually decreases from 8.89 Å to 5.94 Å with increasing V_HBr_%, which confirms that the lattice spacing decreases with increasing Br ions. Moreover, the slope displays an obviously abrupt change from the tetragonal to cubic phase upon increasing V_HBr_% from 10% to 20%. The *a* of CH_3_NH_3_Pb(Br_x_I_1−x_)_3_ exhibits a linear relationship above 20% of V_HBr_%, as shown in Fig. 2. This linear trend satisfies Vegard’s Law^31,32^, and thus the lattice parameter changes linearly with composition of the perovskite. In general, single-phase mixed-halide perovskite CH_3_NH_3_Pb(Br_x_I_1−x_)_3_ crystals were synthesized by the facile solvothermal method.

**Figure 3:**
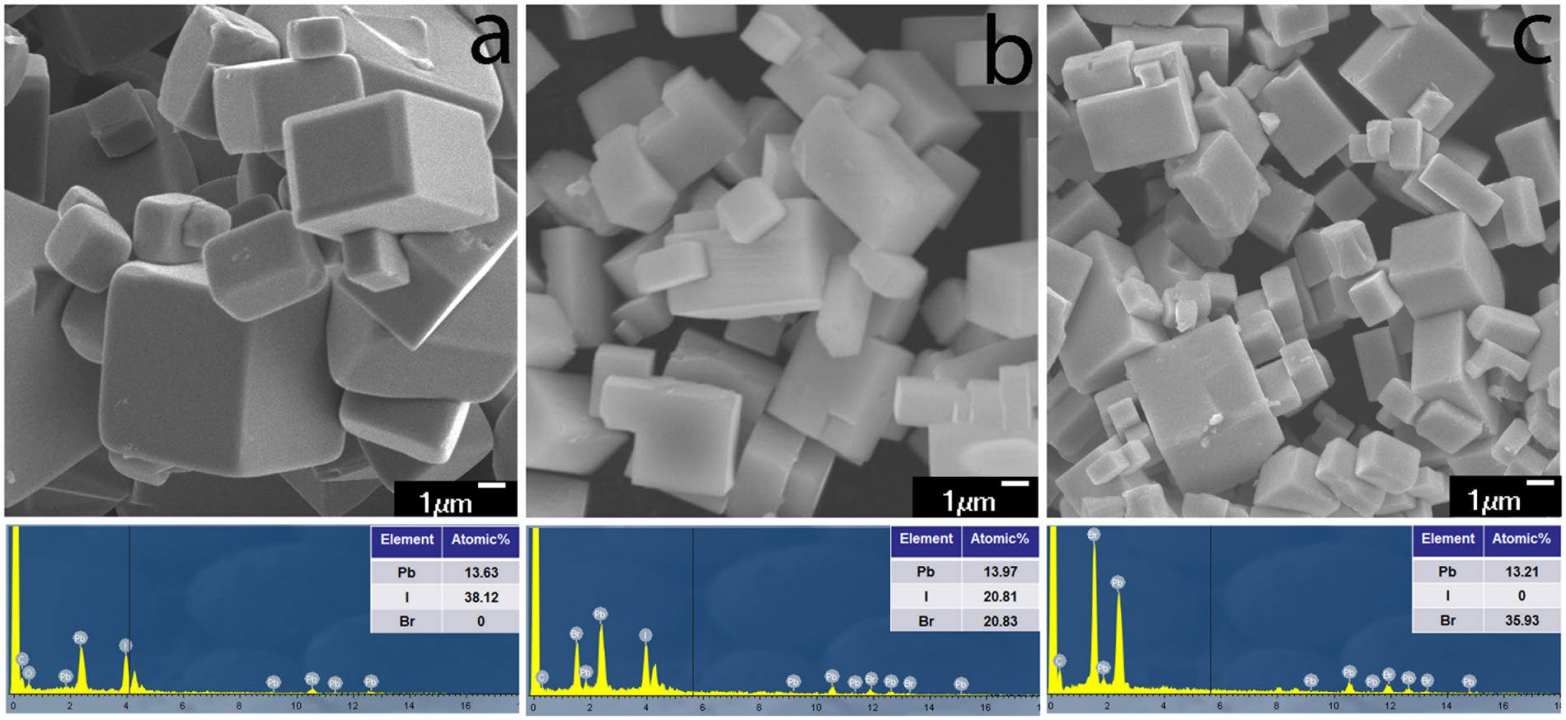
The SEM images of CH_3_NH_3_Pb(Br_x_I_1−x_)_3_ crystals obtained at 150 °C for 4 h with V_HBr_% = 0% (**a**), 50% (**b**), 100% (**c**) and the corresponding EDS spectra.

To further analyse the morphologies and compositions of the CH_3_NH_3_Pb(Br_x_I_1−x_)_3_ crystals, SEM images of crystals with V_HBr_% for 0%, 50%, and 100% and the corresponding EDS spectra are shown in Fig. 3. Furthermore, the morphologies of crystals with others V_HBr_% for 20%, 40%, 60%, and 80% are shown in Fig. S1. The SEM images show that all of the as-synthesized crystals retain cuboid shapes. Length of side of the cuboid shapes become gradually shorten from 3–5 μm to 1–3 μm with increasing the V_HBr_%, which implies the crystal structure variation. The EDS spectra with V_HBr_% for 0%, 50%, and 100% show that the composition ratio of I + Br to Pb is 2.796, 2.98, and 2.719, respectively, which are slightly different from the previous reports^12^, because the iodine or bromine atoms can possibly escape and metallic Pb can separate from the perovskite crystal under the experimental conditions of EDS^33^. The composition ratio of Br to I + Br is 50% in Fig. 3(b), which indicates that V_HBr_% agree well with the predicted × (the composition ratio of Br to I + Br).

To further confirm the compositions of CH_3_NH_3_Pb(Br_x_I_1−x_)_3_ crystals with V_HBr_% from 0 to 100%, we measured the XPS spectra of the crystals, the XPS full scan spectra and detailed spectra of Pb 4f, I 3d and Br 3d are provided in Fig. S2. The average compositions were calculated using the XPS peak areas of I 3d, Br 3d, the area of I 3d shorten and Br 3d increase, which confirm that the composition percent of I lacked and Br increased depending on volume ratio of HBr. Table 1 shows the elemental composition in CH_3_NH_3_Pb(Br_x_I_1−X_)_3_ crystals with increasing V_HBr_% from 0 to 100%, the composition ratio of Br to I + Br increased from 0 to 1. The results again show that the V_HBr_% is nearly equal to the composition ratio of Br to I + Br, that is x. It should be noted that this is the first demonstration of the synthesis of mixed-halide perovskite CH_3_NH_3_Pb(Br_x_I_1−x_)_3_ crystals in the entire composition range via solvothermal method.

**Table 1:** Elemental composition in CH_3_NH_3_Pb(Br_x_I_1−X_)_3_ crystals with V_HBr_%.

V_HBr_%	Br	I	I + Br	Br/(I + Br)	I/(I + Br)	CH_3_NH_3_Pb(Br_x_I_1−X)3_
0	0	38.12	38.12	0	1	CH_3_NH_3_PbI_3_
20	4.18	17.21	21.39	0.19	0.81	CH_3_NH_3_Pb(Br_0.19_I_0.81_)_3_
40	5.13	9.54	14.67	0.35	0.65	CH_3_NH_3_Pb(Br_0.35_I_0.65_)_3_
50	20.81	20.83	41.64	0.5	0.5	CH_3_NH_3_Pb(Br_0.5_I_0.5_)_3_
60	12.26	10.77	23.03	0.53	0.47	CH_3_NH_3_Pb(Br_0.53_I_0.47_)_3_
80	16.10	4.10	20.20	0.79	0.21	CH_3_NH_3_Pb(Br_0.79_I_0.21_)_3_
100	35.93	0	39.93	1	0	CH_3_NH_3_PbBr_3_

**Figure 4:**
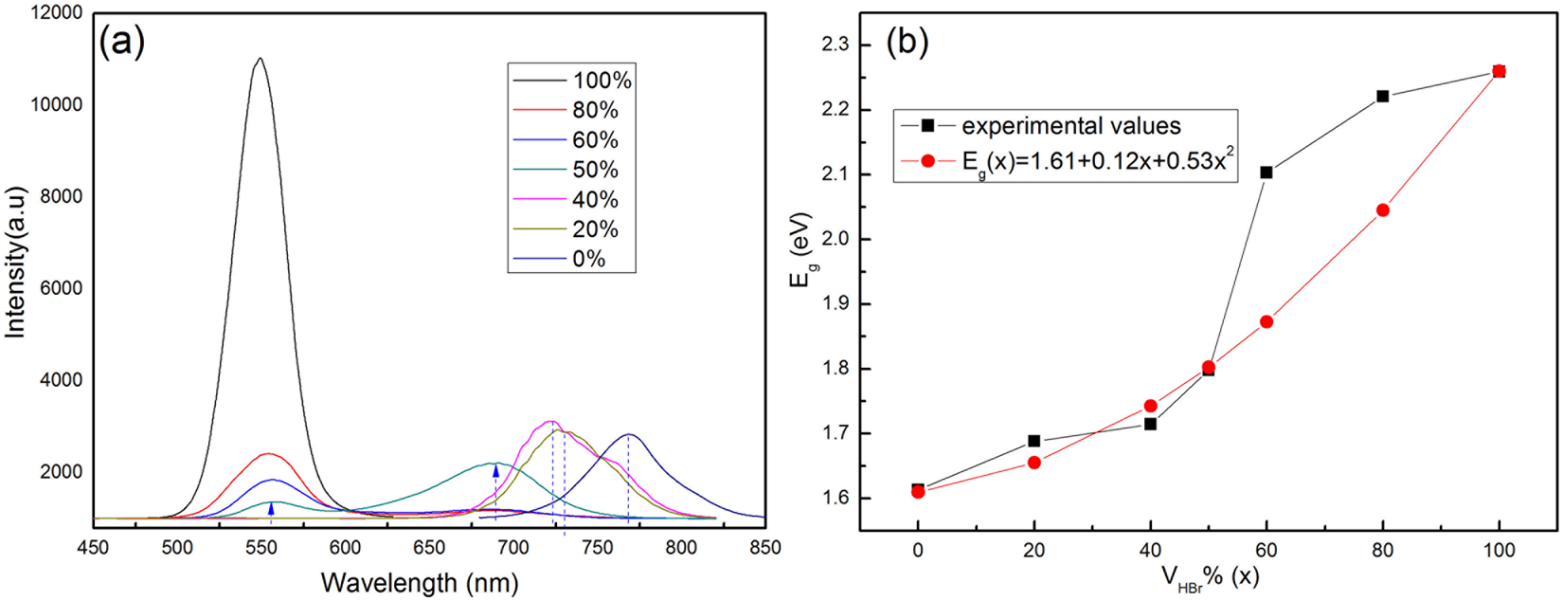
(**a**) The PL spectra of CH_3_NH_3_Pb(Br_x_I_1−x_)_3_ crystals under a low illumination power of 3 mW with V_HBr_% from 0% to 100%; (**b**) the relationship between the band gaps (E_g_) of CH_3_NH_3_Pb(Br_x_I_1−x_)_3_ and V_HBr_%.

The varied of composition could be influenced the band gap or optical properties of CH_3_NH_3_Pb(Br_x_I_1−x_)_3_ crystals, we measured the PL spectra of crystals with increasing V_HBr_%, as shown in Fig. 4(a). The PL peaks of the crystals for pure CH_3_NH_3_PbI_3_ and CH_3_NH_3_PbBr_3_ with V_HBr_% for 0% and 100% point at 768.09 nm and 548.82 nm, respectively, corresponding to bandgaps (E_g_) of 1.61 eV and 2.26 eV. A systematic shift of the PL spectra for CH_3_NH_3_Pb(Br_x_I_1−x_)_3_ to shorter wavelengths was observed with increasing V_HBr_%, which declare that the E_g_ can be tuned from 1.61 eV to 2.26 eV by adjusting the halide content confirming in Table 1. And the colours of the crystals also change correspondingly from dark brown for CH_3_NH_3_PbI_3_ to brown-red for CH_3_NH_3_Pb(Br_x_I_1−x_)_3_ and then to yellow for CH_3_NH_3_PbBr_3_ upon increasing the Br ions, as shown in Fig. S3. Furthermore, the PL spectrum of the crystals at 50% V_HBr_% shows two emission peaks in Fig. 4(a), which imply that the crystal possibly comprises two phases^25^.

The E_g_ variation with V_HBr_% in CH_3_NH_3_Pb(Br_x_I_1−x_)_3_ is plotted in Fig. 4(b). According to previous studies^16^, the E_g_ nonlinear variation with the composition x (V_HBr_%) in the alloy can be expressed by the following quadratic equation (eq. 1):

1 $$\begin{array}{rcl}{{\rm{E}}}_{{\rm{g}}}{[\text{CH}}_{3}{{\rm{NH}}}_{3}{\rm{Pb}}{({{\rm{Br}}}_{{\rm{x}}}{{\rm{I}}}_{1-{\rm{x}}})}_{3}] & = & {{\rm{E}}}_{{\rm{g}}}{[\text{CH}}_{3}{{\rm{NH}}}_{3}{{\rm{PbI}}}_{3}]+{({\rm{E}}}_{{\rm{g}}}{[\text{CH}}_{3}{{\rm{NH}}}_{3}{{\rm{PbBr}}}_{3}]\\  &  & -{{\rm{E}}}_{{\rm{g}}}{[\text{CH}}_{3}{{\rm{NH}}}_{3}{{\rm{PbI}}}_{3}]-{\rm{b}}){\rm{x}}+{{\rm{bx}}}^{2}\end{array}$$

where b is the bowing parameter, which depends on the properties of the inter-substitutional atoms^34^. The bowing parameter illustrates the fluctuation degree in the crystal field or the nonlinear effect arising from the anisotropic nature of binding^35^. A least-squares fit (red line) of the E_g_ in Fig. 4(b) yields bowing parameter of b = 0.53 eV, resulting in eq. 2.

2 $${{\rm{E}}}_{{\rm{g}}}(x)=1.61+0.12\,{\rm{x}}+0.53\,{{\rm{x}}}^{2}$$

The experimental values of E_g_ agree well with values of the least-squares fit below 50%, as shown in Fig. 4(b), which shows that CH_3_NH_3_PbI_3_ and CH_3_NH_3_PbBr_3_ have good miscibility. Above 50%, CH_3_NH_3_PbBr_3_ is predominant in growth of the crystals, which confirms in Table S1.

**Figure 5:**
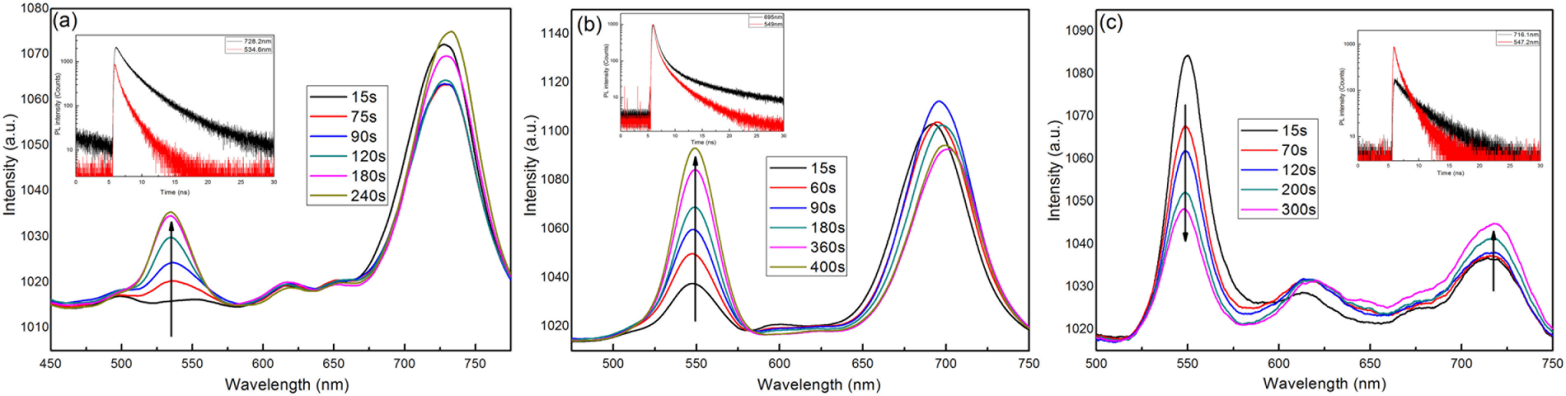
The PL spectra of CH_3_NH_3_Pb(Br_x_I_1−x_)_3_ obtained with V_HBr_% for 40% (**a**), 50% (**b**), and 60% (**c**) after different light-soaking times excited at 3 mW with 375 nm. The inset shows the time-resolved PL (TRPL) dynamics of the two peaks.

To further understand the origin of the PL feature, Fig. 5 shows the PL spectra of the crystals with V_HBr_% for 40%, 50%, and 60% under sequential illumination. Initially, the perovskite with 40% displays an emission peak at 728.2 nm (1.7 eV). With continuous illumination from 15 s to 240 s, a new higher-energy peak forms at 534.6 nm (2.32 eV) and grows in intensity, as shown in Fig. 5(a), which indicates that small clusters enriched in bromine emerge in the crystals and act as recombination centres. Moreover, we should point out that the position of this new peak is independent of the halide composition and bandgap.

Similarly, for crystals with 50%, we found that the emission peak at 549 nm grows greatly in intensity with prolonged illumination, as shown in Fig. 5(b), which ascribe to enriched bromine content. In addition, the emission from the Br-rich region is significantly weaker than that of the iodide-rich region, as shown in Fig. 5(a,b), because charge carriers caused by illumination are quickly transported and accumulated at the iodide-rich region^26^.

When increasing the V_HBr_% to 60%, however, the peak at 547.2 nm decays, and another peak at 716.1 nm grows with prolonged illumination, as shown in Fig. 5(c). The increase in intensity of the lower-band-gap PL peak suggests that these iodide-enriched regions (defects), which act as recombination centre traps, have higher luminescence efficiency than the rest of the perovskite crystals. In other words, I-rich regions serve as the primary charge-carrier recombination sites or irrespective of the carrier generation site in the mixed-halide systems. Similar arguments of charge transfer between Br-rich and I-rich regions as well as trap-initiated recombination have been proposed in earlier studies^25^.

According to reports by Slotcavage^36^ and the experimental results, we speculated that halide phase separation of CH_3_NH_3_Pb(Br_x_I_1−x_)_3_ in small halide-enriched domains is induced by sequential illumination, and the localized strain further promotes halide phase separation, which based on halide migration and possibly caused by photo-excited charge interactions. And the halide migration in perovskites is thought to occur through halogen vacancies^37^. This instability may limit the achievable voltages resulting in degraded performances of related photovoltaic devices.

It is again confirmed that halide phase separation occurs in the time-resolved photoluminescence (TRPL) measurements, as shown in the inset in Fig. 5. The TRPL spectra show that the higher-energy band (red line) decays more rapidly than the lower-energy band (black line), which indicates that the initially formed mixed-halide perovskite, with 40 to 60% bromide content, are comprised of two species or two phases. To testify the range of V_HBr_% for photoinduced phase separation, we have synthesized CH_3_NH_3_Pb(Br_x_I_1−x_)_3_ crystals with different V_HBr_% and treated them with continuous illumination. The results demonstrate that the crystals display phase separation with V_HBr_% from 40% to 60%, as shown in Fig. 5, the crystals with V_HBr_% = 20% and 80% did not display phase separation, as shown in Fig. S4. Moreover, we should state here that the photoinduced change of PL spectra never return to original status after keeping sample for several hours in dark at room temperature, which is inconsistent with previous reports^25,36,38^. It ascribe to that the crystals have the larger crystallite size and higher crystalline quality, which reduce ion migration while enhancing the stability of perovskite materials^39^.

**Figure 6:**
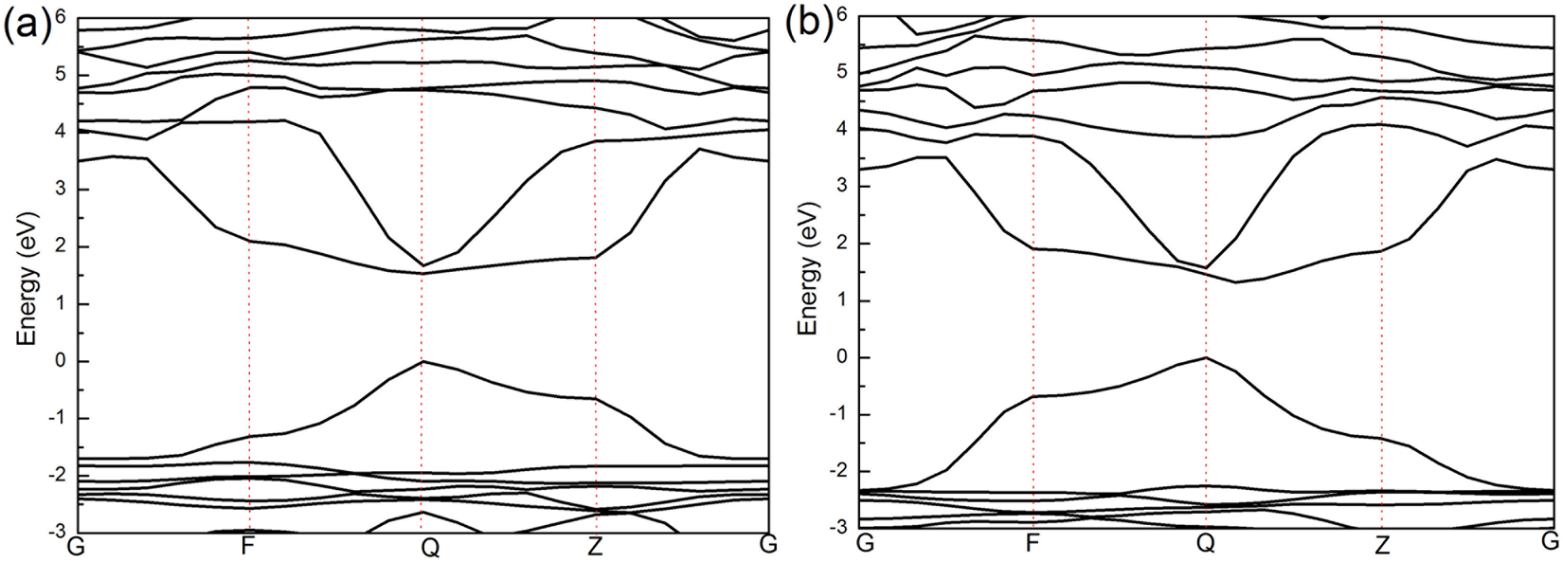
Electronic band structures of CH_3_NH_3_Pb(I_1−x_Br_x_)_3_ with (**a**) x = 0.333 and (**b**) 0.667, Zero energy is set at the top of the valence bands.

Furthermore, the above experimental observations in the optoelectronic properties with various halide contents provide insight into the tuneability of mixed-halide perovskite. To clarify the relationship between the PL feature and the band structure, in this work, we using first-principle calculations study the band structure under variable doped composition conditions, based on experimental lattice parameters. Considering the composition of the unit cell, we focused on the pseudo-cubic phase of the mixed-halide materials of CH_3_NH_3_Pb(Br_x_I_1−x_)_3_ with x = 0.333 and 0.667, and used the band gap approximation of them to fit the result of band gap of CH_3_NH_3_Pb(Br_x_I_1−x_)_3_ crystals with V_HBr_% for 40% and 60%^40,41^. Figures 6 and S5 illustrate the results of the band structure, partial density of states (PDOS) and total density of states (DOS) for the CH_3_NH_3_Pb(Br_x_I_1−x_)_3_ with x = 0.333 and 0.667. It is found that the valence band maximum (VBM) originate mainly from the strong interaction of the Br-4p, I-5p, Pb-6s and Pb-6p states, the conduction band minimum (CBM) is mainly composed of Pb 6p states for x = 0.333 and 0.667. Moreover, the addition of Br introduces Br 4p states in the VBM whereas the VBM mixed contribution from I 5p and Br 4p in x = 0.333 and 0.667. And the Pb 6p contribution at CBM is unchanged at all systems. The results also show that the E_g_ of the bromine doped by 33.3% and 66.7% are 1.53 eV and 1.61 eV in Fig. 6, respectively. However, the range of band gap of the Br doped by 33.3% and 66.7% are 1.53–3.22 eV and 1.61–3.60 eV. Thus, they can be interpreting the PL patterns of the bromine doped by 40% and 60% corresponding with E_g_ values (1.7~2.32 eV and 1.73~2.26 eV, respectively), as shown in Fig. 5(a,c).

In summary, The mixed-halide hybrid perovskite CH_3_NH_3_Pb(Br_x_I_1−x_)_3_ (0 ≤ x ≤ 1) crystals have been systematically synthesized by a solvothermal method through adjusting concentration of Br ions. The XRD indicated that the crystalline structure transitioned from the tetragonal phase to the cubic phase with the introduction of Br ions, and the crystals have higher crystallinities and purities. The SEM showed that all the as-synthesized crystals retain cuboid shapes. Furthermore, PL peaks of the CH_3_NH_3_Pb(Br_x_I_1−x_)_3_ crystals could be tuned from 768 nm to 549 nm, corresponding to a variation in the bandgap from 1.61 eV to 2.26 eV. Moreover, CH_3_NH_3_PbI_3_ and CH_3_NH_3_PbBr_3_ had good miscibility below 50% about V_HBr_%. Notably, CH_3_NH_3_Pb(Br_x_I_1−x_)_3_ (0.4 ≤ x ≤ 0.6) crystals obviously appear phase separation by prolonged illumination due to the formation of small clusters enriched with lower-band-gap, iodide-rich and higher-band-gap, bromide-rich domains, which induced localized strain to promote halide phase separation. In addition, the electronic band structures of the crystals were used to explain many of peaks in the PL patterns with V_HBr_% about 40% and 60%. Meanwhile, modifying the perovskite morphology and crystallinity greatly improved the stability.

## Experiments Methods

### Synthesis

All chemical reagents (analytical grade) were directly used without further purification and were supplied by Sigma-Aldrich.

The similar experimental process used here had been reported in our previous work^33^. Pb(Ac)_2_·3H_2_O (60 mg, Ac^−^ = CH_3_COO^−^, 99.9%) was completely dissolved in a mixed solution of hydroiodic acid (HI, 45% in water) and hydrobromic acid (HBr, 40% in water). Then, 30 mL of isopropanol (IPA, 99.9%) was added and stirred for 5 min, and 0.3 mL of a methylamine solution (CH_3_NH_2_, 30% in water) was added dropwise. The mixture was further stirred for 5 min and then put into 50 mL stainless steel Teflon-lined autoclave, and was sealed and heated in furnace at 150 °C for 4 h, after cooling naturally to room temperature. The precipitates were collected and washed with isopropanol by centrifugation at room temperature, and then were dried under vacuum at 60 °C for 4 h. We mixed the solution in various volume ratios of HI and HBr, and V_HBr_% is defined as the ratio of V_HBr_:V_HI+HBr_ (the total volume of HI and HBr is 1 mL).

### Characterization

The structures of the products were investigated by X-ray diffraction (XRD, X’TRA) using Cu Kα radiation (λ = 0.1542 nm). The X-ray tube voltage and current were set at 40 kV and 40 mA, respectively. The morphologies and elemental analyses of the products were observed by field-emission scanning electron microscopy (FE-SEM, JSM-7000 F) in energy-dispersive spectroscopy (EDS) mode. X-ray photoelectron spectroscopy (XPS, PHI 5000 Versa Probe) was used to identify the elemental compositions of the products, and the resolution of the spectrometer was chosen to be 0.6 eV with a pass energy setting of 40 eV. The photoluminescence (PL) spectra of the products were recorded on a HORIBA iHR 320 fluorescence spectrophotometer with an excitation wavelength of 375 nm at room temperature. The 375 nm line of a picosecond pulsed laser diode (PicoQuant PDL 800-D) was used as the excitation light source for time-resolved PL measurements, and the PL decays were recorded by a time-correlated single-photon counting module and a picosecond event timer (PicoHarp 300)

## Computational Methods

The band structures of the pseudo-cubic phase CH_3_NH_3_Pb(Br_x_I_1−x_)_3_ are calculated within the framework of density functional theory by using the CASTEP package. Norm-conserving pseudopotentials and Perdew-Burke-Ernzerhof (PBE) functional with the generalized gradient approximation (GGA) were used to model the electron-ion interactions and exchange-correlation potential, respectively^42,43^. We focused on the pseudo-cubic phase of the mixed-halide materials of CH_3_NH_3_Pb(Br_x_I_1−x_)_3_ with x = 0.333 and 0.667. The high cutoff energy for the plane-wave basis is set at 750 eV and the Brillouin zone is sampled by a 5 × 5 × 5 *k*-point sampling grid. The convergence tolerance of maximum force, maximum displacement and energy were 0.01 eV/Å, 5.0 × 10^−4^ Å and 5.0 × 10^−6^ eV/atom, respectively. These parameters were controlled to ensure convergence^40^.

## Electronic supplementary material

supplementary information
