# Supplementary material for: Photoluminescence Study of the Photoinduced Phase Separation in Mixed-Halide Hybrid Perovskite CH3NH3Pb(BrxI1−x)3 Crystals Synthesized via a Solvothermal Method
Source: Sci Rep. 2017 Dec 18;7:17695. doi: 10.1038/s41598-017-18110-6 (PMC5735176; doi:10.1038/s41598-017-18110-6)
Supplement: Supplementary file 1 — supplementary information [file 41598_2017_18110_MOESM1_ESM.docx]

**Supporting Information**

**Photoluminescence Study of the Photoinduced Phase Separation in Mixed-Halide Hybrid Perovskite CH_3_NH_3_Pb(Br_x_I_1-x_)_3_ Crystals Synthesized via a Solvothermal Method**

Baohua Zhang^1, 2^, Fuqiang Guo^2^, Junjun Xue^3^, Lianhong Yang^1, 2^, Yafei Zhao^1^, Mei Ge^1^, Qing Cai^1^, Bin Liu^1^, Zili Xie^1^, Dunjun Chen^1^*, Hai Lu^1^, Rong Zhang^1^, and Youdou Zheng^1^

*1* *Key Laboratory of Advanced Photonic and Electronic Materials, School of Electronic Science and Engineering, Nanjing University, Nanjing, 210093, China;*

*2 Department of Physics, Changji College, Changji, 831100, China;*

*3 School of Electronic Science and Engineering, Nanjing University of Posts and Telecommunications, Nanjing, 210023, China.*

** Correspondence to Dunjun Chen, E-mail:* [*djchen@nju.edu.cn*](mailto:djchen@nju.edu.cn)



Fig. S 1 The SEM of CH_3_NH_3_Pb(Br_x_I_1-x_)_3_ obtained at 150 ℃ for 4 h

in V_HBr_%=20%(a),40%(b),60% (c),80%(d), respectively.


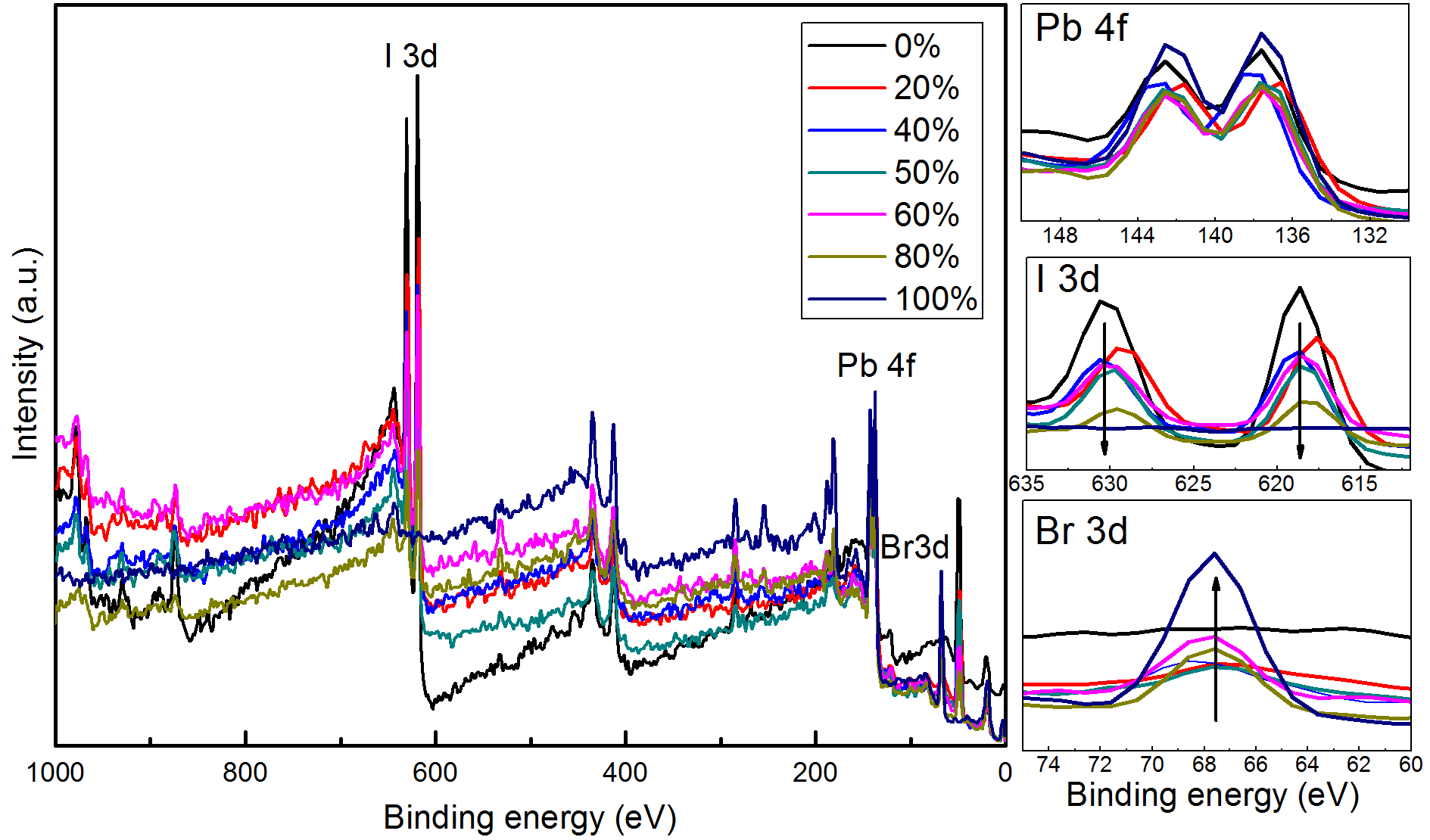
Fig. S 2 XPS spectral of the CH_3_NH_3_Pb(Br_x_I_1-x_)_3_ in V_HBr_% from 0 to 100%,

full scan spectra on the left, detailed spectra of Pb 4f, I 3d and Br 3d on the right


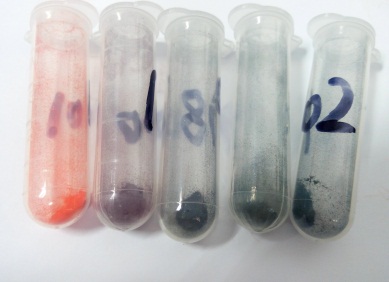

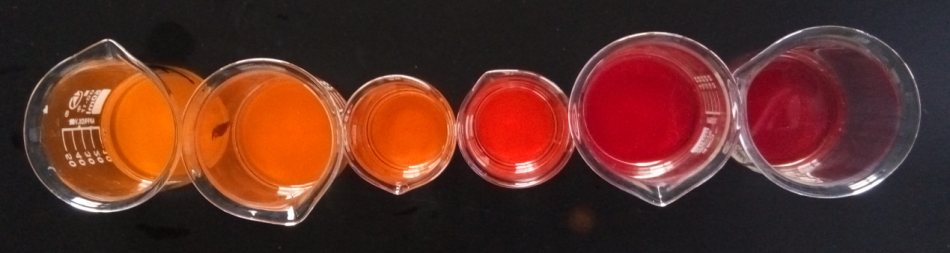


Fig. S 3 The image of CH_3_NH_3_Pb(Br_x_I_1-x_)_3_ obtained at 150 ℃for 4 h in different V_HBr_%

Table S 1 Lattice parameter in CH_3_NH_3_Pb(Br_x_I_1-x_)_3_ crystals with V_HBr_% at different lattice plane

| V_HBr_% | *a*_(100)_ (Å) | *a*_(110)_ (Å) | *a*_(200)_ (Å) |
| --- | --- | --- | --- |
| 20% | 6.21 | 6.12 | 6.22 |
| 40% | 6.15 | 6.06 | 6.14 |
| 50% | 6.05 | 6.06 | 6.09 |
| 60% | 6.02 | 6.02 | 6.01 |
| 80% | 5.95 | 5.95 | 5.95 |
| 100% | 5.93 | 5.94 | 5.94 |


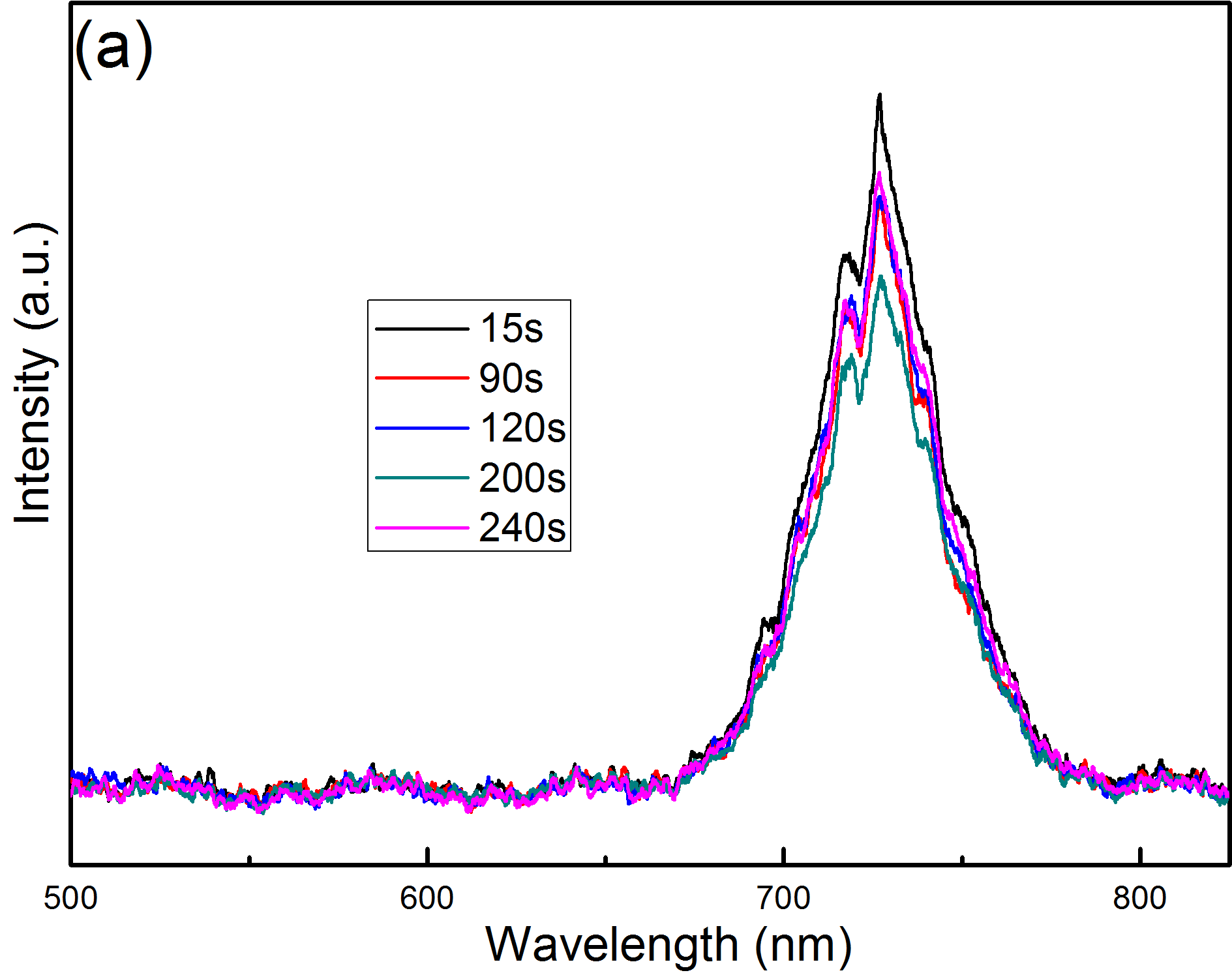

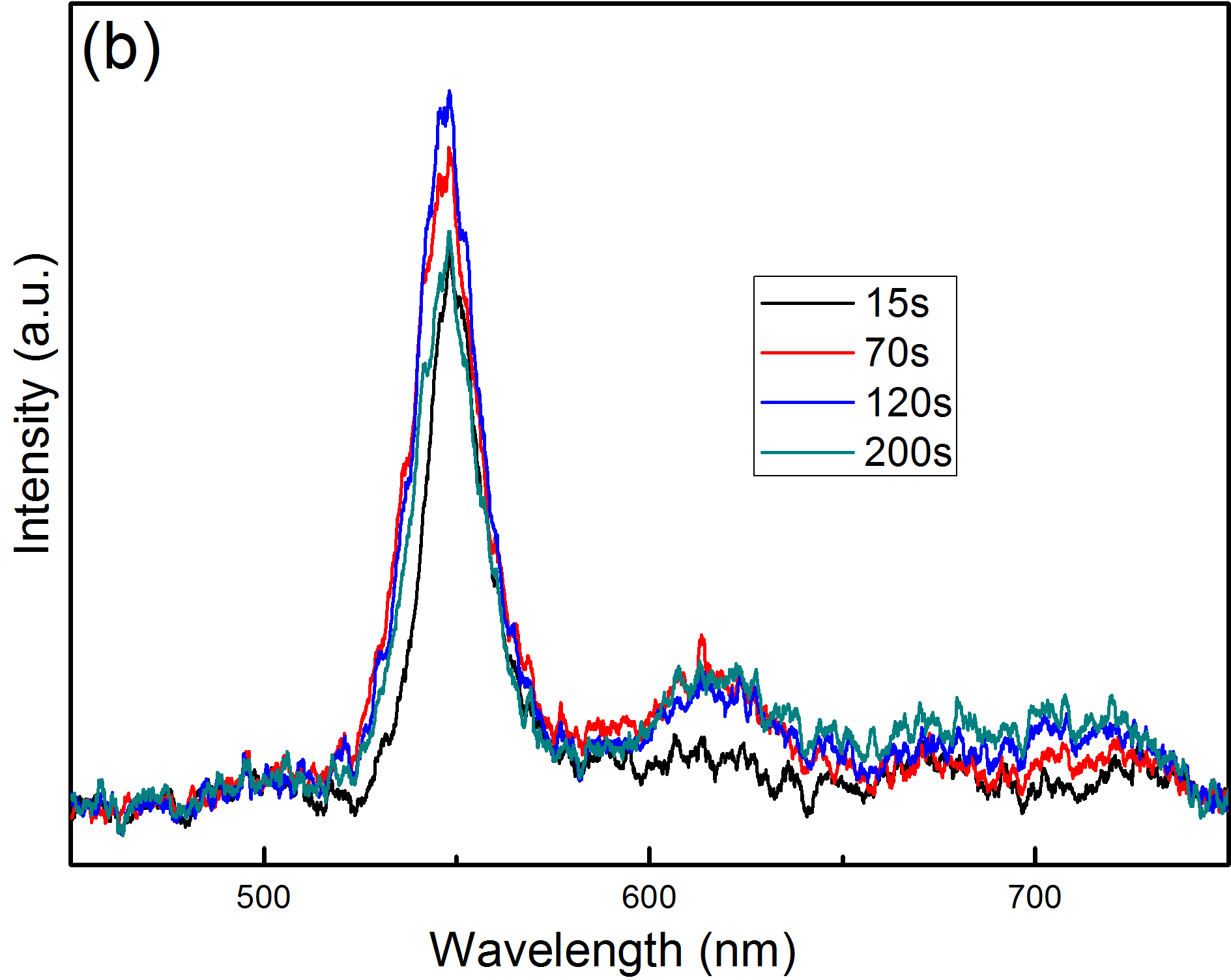


Fig. S 4 the PL spectra of CH_3_NH_3_Pb(Br_x_I_1-x_)_3_ obtained in V_HBr_% = 20% (a) and 80% (b) after different light soaking times excited at 3 mW with 375 nm.


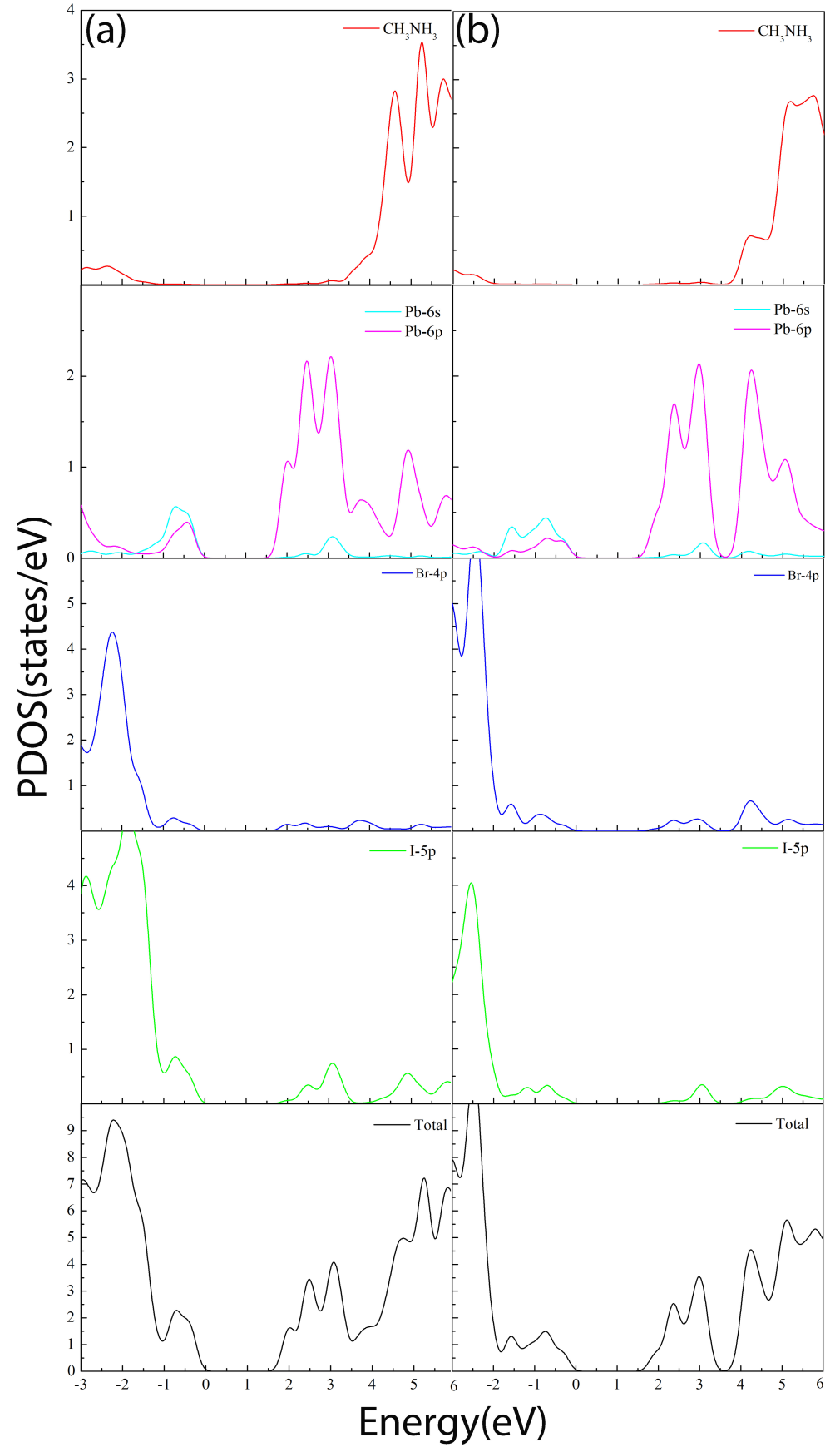


Fig. S 5 Partial density of states (PDOS) and total density of states (DOS)

for the CH_3_NH_3_Pb (Br_x_I_1−x_)_3_ with x= 0.333 and 0.667.
